# Socio-Economic Inequalities in the Prevalence of Multi-Morbidity among the Rural Elderly in Bargarh District of Odisha (India)

**DOI:** 10.1371/journal.pone.0097832

**Published:** 2014-06-05

**Authors:** Pallavi Banjare, Jalandhar Pradhan

**Affiliations:** Department of Humanities & Social Sciences, National Institute of Technology (NIT), Rourkela, Odisha, India; Wadsworth Center, United States of America

## Abstract

**Background:**

Multi-morbidity among elderly is increasingly recognized as a major public health challenge in most of the developing countries. However, information on the size of population suffering from multi-morbidity and socio-economic differentials of multi-morbidity is scarce. The objectives of this paper are twofold; first, to assess the prevalence of various chronic conditions and morbidity among rural elderly and second, to examine the socio-economic and demographic factors that have a significant effect on the morbidity.

**Methods:**

A cross-sectional survey has been done using multi-stage random sampling procedure that was conducted among elderly (60+ years) in Bargarh District of Odisha during October 2011-February 2012. The survey was conducted among 310 respondents including 153 males and 157 females. Descriptive analyses were performed to assess the pattern of multi-morbidity. Logistic regression analyses were used to see the adjusted effect of various socio-economic and demographic covariates of multi-morbidity.

**Results:**

The overall prevalence of multi-morbidity is 57% among rural elderly in Bargarh District of Odisha. The most common diseases in rural areas are: Arthritis, Chronic Obstructive Pulmonary Disease (COPD), High Blood Pressure and Cataract. Results from the logistic regression analyses show that age, state of economic independence and life style indicators are the most important measured predictors of multi-morbidity. Unlike earlier studies, wealth index and education have a marginal impact on multi-morbidity rate. Moreover, the occurrence of multi-morbidity is higher for elderly males compared to their female counterparts, though the difference is not significant.

**Conclusion:**

The high prevalence of morbidity observed in the present study suggests that there is an urgent need to develop geriatric health care services in a developing country like India. Any effort to reorganize primary care for elderly people should also consider the high prevalence of multi-morbidity among rural elderly in India.

## Introduction

The world is moving towards population aging. It is projected that by the year 2020, there will be one billion elderly people (65+ years) in the world and 71% of whom will live in low-income countries [1]. Elderly population in India is approximately hundred million forming 10% of the total population [2], [3]. The report by Integrated programs for older person in 2008 by the Ministry of Social Justice and Empowerment (Government of India 2008) reveals that the number of people in the 60+ age group in India will increase to 198 million by 2030 [4]. However, the progression of aging leads to loss of adaptive response towards stress and growing risk of age related diseases, resulting in progressive increase in age specific mortality. From morbidity point of view, at least 50% of the elderly in India have chronic diseases [2]. This implies that aging population will suffer from chronic medical conditions and the prevalence of multiple chronic conditions is expected to increase [5]. Many studies have been carried out on the prevalence of multi- morbidity in Europe [6], [7], [8], the Middle East [9], Australia [10], the United States [5], [11], [12], Bangladesh [13] and Canada [14], [15], [16]. However the available literature reveals limited studies on multi-morbidity amongst elderly people in developing countries. In Indian context few studies on prevalence of multi-morbidity have been conducted [17], [18]. Multi-morbidity becomes progressively more common with age [19], [20], [21], [22] and is associated with high mortality [23], reduced functional status [24], [25], and increased use of both inpatient and ambulatory health care [5].

Although, the association between socioeconomic status and prevalence of individual chronic diseases is well established [26], [27] few studies have examined the association between multi-morbidity and socio-economic status [20], [21], [28]. Another set of studies have investigated how diseases distribute or co-occur in the same individual. Several studies have used different approaches to address these issues [23], [16]. A study conducted in Australia found that 85% of 70+ year elderly have multi-morbidity and the prevalence is higher among elderly with obesity, elderly female, elderly with low socioeconomic status, elderly living alone and less educated [20].

A nested case–control study of general practitioners in South Netherlands Community residents found that multi-morbidity was highly correlated with increasing age, low socioeconomic status, and those who had diseases prior to the study [29]. A small number of studies have identified the relationship between multi-morbidity, disability and functional decline. However, study among the Spanish elderly found out that multi-morbidity was associated with impaired functioning [30]. In contrast another study found that multi-morbidity was not associated with physical activity levels [31]. Landi et al. (2010) studied on Italians living in a community and concluded that multi-morbidity affected 4-year mortality, only if associated with disability [32]. A research on residential volunteers in Hong Kong concludes that depression prevalence was associated with the number of chronic conditions [33]. Walker. (2007) conducted a study on multi-morbidity with healthcare utilization and quality of life among Australian general population. He found that persons with 3 or more chronic conditions were more likely to feel distressed or pessimistic about their lives [20]. Wolff et al. (2002) concluded that increasing number of diseases increases hospitalizations, preventable complications, and expenditures [5].

Most of the available studies on multi-morbidity in India are disease specific and fail to provide comprehensive overview of wide range of diseases occurring among rural elderly. One of the studies in Chandigarh found that elderly female were more prone to morbidity [34], [35]. Another study on multi-morbidity among elderly in Karnataka, found that the prevalence of multi-morbidity was equally distributed among both men and women [35]. A study conducted by Shankar et al., found that the common morbidity among Indian elderly is Arthritis with overall prevalence of 57.08%, followed by Cataract (48.33%), Hypertension (11.25%) [36]. But the prevalence of old age related morbidities have increased with advancing age. Variables like caste, literacy and socioeconomic status did not show significant association with the prevalence of multi-morbidity [36].

Looking at the growing concern on multi-morbidity in India, there is necessity of better understanding of the epidemiology of multi-morbidity to develop interventions to prevent it and align health care services more closely for the rural elderly patients’ needs. So, an intensive study on multi-morbidity among rural elderly is necessary to address the multiple deprivation of health to reduce the health burden among elderly. The objectives of this paper are two fold; first, to assess the prevalence of various chronic conditions (ICD 10) and morbidity among rural elderly in Bargarh district of Odisha and second, to examine the socio-economic and demographic factors that have a significant effect on the morbidity.

## Data and Methods

### Ethics Statement

The study was conducted in Bargarh district of Odisha, India. The study aims to explore the familial setups, roles, health status and expectations of the elderly. Before collecting necessary information from selected elderly, following consent form was signed by the respective respondent:

*“I am going to ask you some personal questions that some of the people find difficult to answer. Your answers are completely confidential, your name, will not be disclosed to anyone, and will never be used in connection with any of the information you tell me. You do not have to answer any questions that you do not feel comfortable, and you may withdraw from this interview at any time you want to. However, your answers to these questions will help us to understand the senior citizens situation. We would greatly appreciate your help in responding to this interview. Would you be willing to participate?”*

If the respondent provided consent, an interview was conducted.

The study was approved by the Doctoral Research Committee (DRC) of National Institute of Technology, Rourkela, Odisha, India.

### Sample Selection

A cross-sectional survey using multi-stage random sampling procedure was conducted among elderly (60+ years) in Bargarh District of Odisha during October 2011-February 2012. Selection of respondents involved three stages of sampling procedure. Block was selected at the first stage. Then village was selected at the second stage followed by selection of target respondents at the third stage. The targeted sample size was 320. Data were collected by face-to-face interviews with a pre-tested structured questionnaire. Ten respondents who were extremely frail could not respond to the questionnaires. So, finally 310 respondents were considered for analysis resulting in a response rate of 97%.

As per Census 2001, there are 12 blocks in Bargarh i.e. Bargarh, Barpali, Attabira, Bheden, Sohella, Bijepur, Padmpur, Gaisilet, Paikmal, Jharbandh, Ambabhona and Bhatli. Two blocks namely Sohella and Padmpur were selected randomly. Twenty respondents (10 Male and 10 Female) were selected from each village. So, 16 villages (10 from Sohela and 10 from Padampur) were selected to get the required number of respondents. Villages were selected using probability proportion to sample size (PPS). At the village level, a sampling framework was prepared separately for male and female respondents. A complete listing of the households in a selected village was done. During the listing in each household, all the members aged 60+ were listed. Each member’s actual age and gender were noted. Accordingly, 10 Male and 10 Female elderly were selected randomly.

### Dependent Variables

In this paper morbidity has been taken as dependent variable. In order to determine the occurrence of morbidities, respondents were asked, *“Has a doctor or nurse ever told you that you have any of the following ailments viz; Arthritis, Cerebral embolism, Stroke or Thrombosis, Angina or heart disease, Diabetes, Chronic lung disease, Asthma, Depression, High blood pressure, Alzheimer’s disease, Cancer, Dementia, Liver or Gall bladder illness, Osteoporosis, Renal or Urinary tract infection, Cataract, Loss of all natural teeth, Accidental injury (in past one year), Injury due to fall (in the past one year), Skin disease, and Paralysis?”.*

For descriptive analysis, we have categorized the prevalence of morbidity into four groups: 1) elderly having no morbidity, 2) elderly having one morbidity, 3) elderly having two morbidities & 4) elderly having three or more morbidity. Multi-morbidity is defined as those who are having 2 or more morbidities. For logistic regression, morbidity was recorded into binary form i.e. elderly having one or no morbidity was taken as ‘0’ and one having 2 or more morbidity i.e. multi morbidity was taken as ‘1’.

### Independent Variables

Various socio-economic and demographic factors are treated as independent variables namely a) Age (in five years age groups), b) Sex, c) Marital status, d) Education, e) Wealth quintile, f) Caste, g) State of economic dependence, h) Living arrangement, and i) Life style indicators.

The demographic variables which have been considered are: a) Sex divided into two categories (1. female 2. male), b) Age group (in five years group) divided into four categories (1. 60–65 years 2. 65–70 years 3. 70–75 years 4. 75+ years).

The role of marital status has been clearly demonstrated in the literature examining the relationship between marital status and health outcomes [39]. All of the various unmarried states (being single, never married, being separated/divorced and being widowed) have been associated with elevated mortality risks [40]. It has been proved that married people are better-off in health and suffer from less morbidity. In this study, marital status has been classified into two categories viz., 1) currently married, 2 widowed/divorced or separated. Educational qualification is divided into four categories - 1. No formal education, 2. Primary school and less completed 3. Primary school completed 4. Secondary school and above completed.

The questionnaire also has questions related to thirty three assets owned by households which were later converted into wealth quintile or wealth index. The wealth index is based on household assets and housing characteristics, such as (mattress, pressure cooker, chair, bed, table, electric fan, radio, black and white television, color television, sewing machine, mobile phone, any other phone, computer, refrigerator, watch, bicycle, motorcycle, animal drawn cart, car, water pump, thresher, tractor and electricity). Using principal component analysis these assets and their characteristics were combined into a single variable. After ranking this variable from low to high, households were divided into five equal-sized groups namely - 1) Poorest (Q1) 2) Poorer (Q2) 3) Middle (Q3) 4) Richer (Q4) 5) Richest (Q5). Caste is divided based on caste schedule followed as per Government of India guidelines - 1. Scheduled Caste/Scheduled Tribe 2. Other Backward Caste 3. General. The state of economic dependence is divided into three categories 1. Not depending on others, 2. Partially dependent 3. Fully dependent.

Living arrangements refers to the type of family in which the elderly live, the headship they enjoy, the place they stay in and the people they stay with, the kind of relationship they maintain with their kith and kin, and the extent to which they adjust to the changing environment [37], [38]. While dealing with the welfare of any specific group, it is important to study their pattern of living arrangement. There exists several living patterns for the elderly such as - living with the spouse, living with children, living with other relations and non-relations and living alone (as an inmate of old age homes). In this study living arrangement is categorized into four categories i.e. 1) living alone, 2) living with spouse/son/daughter, 3) living with spouse and unmarried sons, 4) living with spouse and married son.

A report by US National Cancer Institute in 2002 reveals that the Asian people have been using tobacco in various forms since ages [41]. Moreover, the International Agency for Research on Cancer in 2007 [42] strongly expresses that SLT (smokeless tobacco) is common in Asian countries such as India, Pakistan and Bangladesh. The use of SLT varies by age, sex, ethnicity and socioeconomic status, both within and among countries [43]. A study by Accortt. et.al. (2002) concluded that use of tobacco as well as SLT leads to chronic heart diseases [44].

In this study, we have considered a set of variables as risk behaviors like i) Smoking (1. Yes 2. No), ii) Consumption of alcohol (1. Yes 2. No), iii) Chewing tobacco (1. Yes 2. No).

At first, descriptive analysis was done to assess the socio-economic differentials in the prevalence of multi-morbidity. Secondly, binary logistic regressions were carried out to explore factors responsible for the prevalence of multi-morbidity among rural elderly in Odisha.

Logistic regression can be used to predict a dependent variable on the basis of independents and to determine the per cent of variance in the dependent variable explained by the independents; to rank the relative importance of independents; to assess interaction effects; and to understand the impact of covariates. Logistic regression applies maximum likelihood estimation after transforming the dependent into a logit variable (the natural log of the odds of the dependent occurring or not). So, logistic regression estimates the probability of certain event whether occurring or not. The multiple logistic models can be noted as:
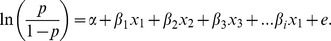
Where, 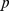 is the probability of occurrence of multi-morbidity, 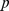 *(y = 1);* 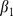, 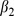, 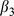,… 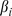 refers to the beta coefficients; 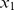, 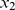, 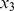, …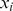 refers to the independent variables and e is the error term.

In all, four models have been applied with different categories of independent covariates (Table 1). SPSS V 20 is used to analyze the data. The survey data was analyzed using descriptive and logistic regression analysis.

**Table 1:** Model design for logistic regression analysis.

Models	Model 1	Model 2	Model 3	Model 4
Variables	Only demographic variables	Only Socio-economic variables	Only life style indicators	All independent covariates
	• Age• Sex• Marital status	• Education• Wealth Index• Caste• State of economic dependence• Living arrangements	• Smoking• Consuming tobacco	• Age• Sex• Marital status• Education• Wealth Index• State of economic dependence• Living arrangements• Smoking• Consuming tobacco

## Results

### Socio-economic and Demographic Profiles of Respondents

Table 2 presents the sample characteristics of the studied population by selected socio-economic covariates. Out of the total sample of 310 respondents, 153 are male and 157 are female. The married people comprise of 60.3% and widowed/divorced or separated comprise of 39.7% of the total sample. Study on Literacy or Education of the respondents’ shows that about 60.3% have no formal education, followed by 27.7% who have completed primary education or less and only 4.5% have completed their secondary school and above. In State of Economic Dependence, about 46.5% are partially dependent, followed by not dependent on others (42.3%) and 11.3% are fully dependent on their spouse, son or other relative. While analyzing Caste structure, Other Backward Caste have the highest share of 57.1%, followed by Scheduled Caste/Scheduled Tribe with 31.9% and General have 11% only. Elderly living with spouse and married son are the most with about 54.5%, followed by living with either spouse/son or daughter and elderly living alone are the least with only 7.7% share. About 58.1% of the population have Below Poverty Line card. About 63% of the respondents are consuming tobacco, 31% of them are used to smoking and a small proportion (4%) in drinking alcohol.

**Table 2:** Percentage distribution of respondents by selected socio-economic characteristics by Gender.

Covariates	%	N
**Sex**		
Male	49.4	153
Female	50.6	157
**Age of the respondents**		
60–65 Years	30.6	95
65–70 Years	35.5	110
70–75 Years	20	62
75 & Above	13.9	43
**Marital Status**		
Currently married	60.3	187
Widowed/Divorced or Separated	39.7	123
**Education status of respondents**		
No formal education	60.3	187
Less than primary	27.7	86
Primary school completed	7.4	23
Secondary school and above	4.5	14
**Wealth quintile**		
Poorest	19.7	61
Poorer	19.4	60
Middle	21	65
Richer	19.7	61
Richest	20.3	63
**Caste**		
General	11	34
Scheduled Caste/Scheduled Tribe	31.9	99
Other Backward Caste	57.1	177
**State of economic dependence**		
Not dependent	42.3	131
Fully dependent	11.3	35
Partially dependent	46.5	144
**Living arrangements**		
Living alone	7.7	24
Living with spouse/Son/Daughter	25.5	79
Living with Spouse and unmarried son	12.3	38
Living with Spouse and married son	54.5	169
**BPL card holder**		
Has the card	58.1	180
**Risk Behaviors**		
Smoking (Yes)	31	96
Consuming Alcohol (Yes)	4.19	13
Consuming Tobacco (Yes)	63.2	196
**N**		**310**

### Prevalence of Morbidity by Gender

Table 3 presents percentage of respondents having selected morbidities by gender. The individuals were asked whether the doctor had ever told them that they might be having any of the above mentioned chronic diseases. To verify the responses, the test results/doctor’s prescriptions/supporting documents were checked during the interview session. This table clearly shows that the most common disease in this rural setup is Arthritis with total 52.9% and it is slightly higher for females with 54.7% of the total sample. A high prevalence of arthritis/joint pain in the current study especially among females was also reported in other studies [45], thus it reflects the hard life faced by women who never retire from household work unless totally disabled.

**Table 3:** Percent of respondents having selected morbidities by Gender.

Morbidities	Male (N = 153)	Female (N = 157)	Total (310)
Arthritis	50.9	54.7	52.9
Cerebral-embolism, stroke or Thrombosis	0.6	1.9	1.2
Heart disease	0.6	4.4	2.5
Diabetes	7.8	10.8	9.3
Chronic obstructive pulmonary disease	30.0	10.1	20.0
Asthma	9.1	10.1	9.6
Depression	7.1	4.4	5.8
High blood pressure	26.1	12.7	19.3
Alzheimer’s disease	3.9	9.5	6.6
Cancer	0.0	1.9	0.9
Dementia	4.5	7.6	6.1
Liver or gall bladder illness	4.5	3.1	3.8
Osteoporosis	1.9	3.1	2.5
Renal or Urinary tract infection	9.1	3.8	6.4
Cataract	21.5	15.9	18.7
Loss of all natural teeth’s	4.5	7.0	5.8
Accidental injury (in past one year)	11.7	6.3	9.0
Injury due to fall (in past one year)	3.9	2.5	3.2
Skin disease	6.5	7.0	6.6
Paralysis	8.4	4.4	6.4

Next prevailing disease followed by Arthritis, with about 20% of the elderly reported was Chronic Obstructive Pulmonary Disease (COPD), with males having a higher share of 30% in comparison to females having just 10.1%. Globally, COPD is expected to rise to the 3rd position as a cause of death and at the 5th position as the cause of loss of disability adjusted life years (DALYs), according to the baseline projections made in the Global Burden of Disease Study (GBDS) by 2020 [46]. Tobacco smoking remains the most important risk factor identified as the cause of COPD and chronic respiratory morbidity [47]. Tobacco related mortality is estimated to be highest in India, China and other Asian countries [48].

The third prevalent morbidity is High Blood Pressure or Hypertension. The result shows that about 19.35% of respondents are suffering from Hypertension. Studies from Karnataka and Kolkata have also reported that the prevalence of hypertension was about 30.5% and 40.5% respectively [49], [50]. The difference in prevalence levels may be due to different geographical factors and may be due to differences in dietary pattern. Cataract is also one of the important morbidities present in the rural population in the studied villages i.e. 18.70%. It is more common in females compared to their male counterparts. Cataract is found to be more common in rural population, which may be due to increased exposure to ultraviolet radiation during long hours of work in open fields [51]. Eighty percent of this blindness is due to cataract alone [52]. Skin diseases, paralysis and accidental injury are also the other forms of morbidities occurring among rural elderly in Odisha.

While comparing the prevalence of disease amongst males and females, it shows that arthritis is more common among females than males, whereas chronic lung disease and high blood pressure are more common among males. Similarly, dementia and Alzheimer’s disease are more common among females and cataract amongst males. For other diseases, both male and females shared similar patterns with slight variations.

### Pattern of Multi-morbidity

**Figure 1:**
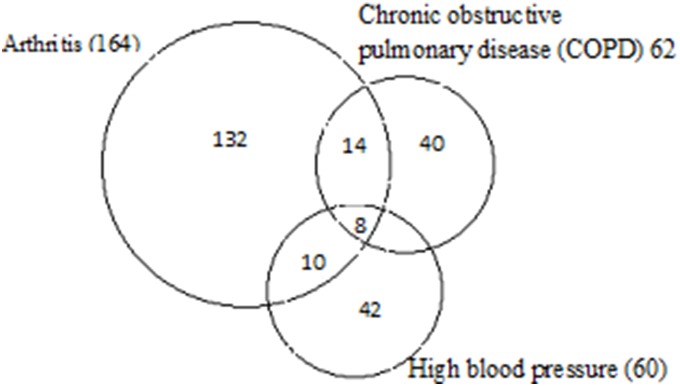
Venn diagram displaying the overlapping of multi-morbidity patterns in numbers related to the total population.

The following Venn diagram (figure 1) shows the overlapping of major morbidities found among rural elderly in Odisha. The three common morbidities are arthritis (164), chronic obstructive pulmonary disease (62) and high blood pressure (60). Amongst 164 elderly people having arthritis about 62 (37%) are suffering from chronic obstructive pulmonary diseases, 60 (36%) are having high blood pressure and (8) 5% are having all the three morbidities.

Hence, the result shows that the occurrence of multi-morbidities is very common among our study population.

### Prevalence of Multi-morbidity by Age Groups

Table 4 shows the relationship between age groups (60–65 years, 65–70 years, 70–75 years and 75+ years) and the intensity of morbidities. The occurrence of morbidities is classified into four groups - i) no morbidity, ii) having one morbidity, iii) having 2 morbidities and iv) having 3 or more morbidities. Multi-morbidity is defined as persons having two or more morbidities. Results from table 4 clearly suggest that, the rate of multi-morbidity increases with the increased age. The rate of multi-morbidity is 74% among 75+ year elderly compared to 40% for 60–65 years age group elderly. Another interesting finding of this study revealed that about 95% of the elderly (in the age group of 75+ years) have at least one morbidity.

**Table 4:** Prevalence of morbidity by age groups.

Number of morbidities	% of respondents by morbidity profile				
	Age group				
	60–65 years	65–70 years	70–75 years	75+years	Total
No morbidity	16.8	9.1	6.5	4.7	10.3
One morbidity	43.2	33.6	24.2	20.9	32.9
Two morbidity	17.9	28.2	35.5	30.2	26.8
Three or more morbidity	22.1	29.1	33.9	44.2	30.0
At least two morbidities (Multi-morbidity)	40.0	57.3	69.4	74.4	56.8
**N**	**95**	**110**	**62**	**43**	**310**

### Socio-economic Differentials in Multi-morbidity

As reviewed in earlier section, the rate of multi-morbidity varies with selected socio-economic and demographic covariates. Results from table 5 shows that the overall prevalence of multi-morbidity was 56.8% among rural elderly in Odisha, similar to what is frequently reported from many developed and developing nations e.g. 53.8% in Bangladesh [13], 55% in Swedish elderly [8], 75% in Australia [10], 65% in North America [11], although the criteria or definition were not identical in those studies. Unlike earlier studies the rate of multi-morbidities was higher for male compared to their female counterpart. This could be partly due to the response bias, as male are more open to disclose their disease experience compared to their female counterparts., Several recent studies revealed that the gender differences in multi-morbidity prevalence are marginal [54]. Many other studies on morbidity also found a strong positive relationship between age and multi- morbidity [55], [10], [11].

**Table 5:** Multi-morbidity prevalence by selected socio-economic and demographic covariates.

Covariates	%	N
**Sex**		
Female	50.3	157
Male	63.4	153
**Age of the respondents**		
60–65 Years	40.0	95
65–70 Years	57.3	110
70–75 Years	69.4	62
75 Years & Above	74.4	43
**Marital Status**		
Currently married	57.8	187
Widowed/Divorced or Separated	55.3	123
**Education status of respondents**		
No formal education	56.7	187
Less than primary	57.0	86
Primary school completed	56.5	23
Secondary school and above	57.1	14
**Wealth quintile**		
Poorest	60.7	61
Poorer	53.3	60
Middle	52.3	65
Richer	63.9	61
Richest	54.0	63
**Caste**		
General	58.8	34
Scheduled Caste/Scheduled Tribe	48.5	99
Other Backward Caste	61.0	177
**State of economic dependence**		
Not dependent	48.1	131
Fully dependent	71.4	35
Partially dependent	61.1	144
**Living arrangements**		
Living alone	54.2	24
Living with spouse/Son/Daughter	59.5	79
Living with Spouse and unmarried son	42.1	38
Living with Spouse and married son	59.2	169
**BPL card holder**		
Yes	58.1	180
No	41.9	130
**Smoking**		
Yes	60.4	96
No	55.1	214
**Consuming Tobacco**		
Yes	60.7	196
No	50.0	114
**N**	**56.8**	**310**

The relationship between economic status (measured in terms of wealth index) and occurrence of multi-morbidity is very weak. The prevalence of multi-morbidities by categories of educational status is identical, revealing the fact that occurrence of diseases are independent of education. Elderly belonging to Other Backward Caste (61%) are more prone to multi-morbidity compared to General Caste (58.8%) and Scheduled Caste/Scheduled Tribe (48.5%) elderly. State of economic independence is strongly associated with the rate of multi-morbidity. The multi-morbidity prevalence is about 71.4% for elderly who are fully dependent on others compared to elderly who are not dependent on others (48.1%). The disease prevalence is lower among elderly those who stay with their spouse and unmarried sons (42.1%) compared to their counterparts. As established in other studies, in this study too, life style indicators are positively associated with the occurrence of multi-morbidity.

### Multivariate Logistic Regression Analysis

Since several of demographic, socio-economic and life style factors are interrelated, multivariate regression models of multi-morbidity are estimated to assess the independent effects of these factors on the occurrence of multi-morbidity, controlling for other predictors in the model. Table 6 presents the results of logistic regression analysis taking four models into consideration.

**Table 6:** Results of logistic regression analysis of factors associated with multi morbidity.

Variables	Model 1	Model 2	Model 3	Model 4
	OR (95% CI)	OR (95% CI)	OR (95% CI)	OR (95% CI)
**Sex**				
Female	1.00			1.00
Male	1.39 (0.85–2.29)			1.68 (0.91–3.11)
**Age**				
60–65 years	1.00			1.00
65–70 years	2.04* (1.16–3.58)			2.33* (1.22–4.45)
70–75 years	3.43** (1.69–6.94)			4.91** (2.18–11.05)
75+years	4.27** (1.87–9.73)			4.65** (1.87–11.52)
**Marital status**				
Currently married	1.00			1.00
Widowed/Divorced or Separated	0.79 (0.47–0.133)			0.92 (0.47–1.78)
**Wealth Index**				
Poorest		1.00		1.00
Poorer		0.93 (0.43–2.02)		1.22 (0.52–2.84)
Middle		0.64 (0.28–1.47)		0.70 (0.28–1.72)
Richer		1.08 (0.47–2.46)		1.41 (0.57–3.48)
Richest		0.59 (0.24–1.43)		0.60 (0.23–1.54)
**Education**				
No formal education		1.00		1.00
Less than primary		1.22 (0.68–2.20)		1.38 (0.69–2.75)
Primary school completed		0.94 (0.37–2.39)		1.62 (0.54–4.89)
Secondary school and above		1.68 (0.49–5.75)		2.36 (0.54–10.35)
**Caste**				
General		1.00		1.00
Scheduled Caste/Scheduled Tribe		0.60 (0.25–1.42)		0.58 (0.22–1.54)
Other Backward Caste		1.02 (0.45–2.32)		0.891 (0.35–2.21)
**State of Economic independence**				
Not depending		1.00		1.00
Fully dependent		3.06* (1.29–7.24)		5.21** (1.99–13.60)
Partially dependent		2.05** (1.20–3.50)		3.02** (1.57–5.81)
**Living arrangement**				
Living alone		1.00		1.00
Living with spouse or son or daughter or anyone		1.44 (0.53–3.93)		1.35 (0.41–4.46)
Living with Spouse and unmarried son		0.64 (0.20–2.00)		0.40 (0.10–1.56)
Living with Spouse and married son		1.55 (0.57–4.20)		1.25 (0.40–3.86)
**Smoking**				
No			1.00	1.00
Yes			1.46* (0.87–2.46)	1.85* (0.98–3.50)
**Chewing Tobacco**				
No			1.00	1.00
Yes			1.72** (1.05–2.81)	2.82** (1.51–5.24)
**Total**				
Constant	−.481	−.183	−.185	−2.212

*significant at 5 per cent level;

**significant at 1 percent level.

Results from Model 1 indicate that among demographic variables, age has a very large effect on the occurrence of multi-morbidity. The prevalence of multi-morbidity increases steadily with age. The Odds Ratio (OR) of multi-morbidity prevalence is about 4.27 (CI: 1.87–9.73) times higher for elderly above 75 years compared to those in 60–65 years age group.

Model 2 assesses the cumulative impact of various socio-economic covariates on multi-morbidity. Results from the analysis shows that among socio-economic variables, only the state of economic independence has significant impact on multi-morbidity. The prevalence of multi-morbidity is significantly higher for the elderly who are dependent on others compared to their counterparts.

Life style indicators (smoking and chewing tobacco) have a significant effect on the occurrence of multi-morbidity (Model 3). The elderly consuming tobacco are 1.72 times more prone to morbidity than those who do not consume tobacco at all. Similarly, elderly who smoke regularly are about 1.46 times more prone to morbidity than those who do not smoke.

Finally, in Model 4 all variables are included to assess the adjusted effect of various demographic and socio-economic covariates on multi-morbidity.

Even after controlling all the covariates - like age and state of economic independence the life style indicators have retained their significant effect on the occurrence of multi-morbidity.

## Conclusions

Given the increasing prevalence of multi-morbidity, understanding the socio-economic differentials in multi-morbidity among rural elderly is important to help national and sub-national health planners to address the issues in a broader perspective. The overall prevalence of multi-morbidity is 57% among rural elderly in Bargarh District of Odisha this fits well with the reporting range of multi-morbidity rates in elderly population [11], [18], [55], [53], [56]. The most common diseases in rural set-up are - Arthritis, COPD, High Blood Pressure and Cataract. Results from the multivariate analysis show that age, state of economic independence and life style indicators are the most important measured predictors of multi-morbidity. Unlike earlier studies, wealth index and education have a marginal impact on multi-morbidity rate. Moreover, the occurrence of multi-morbidity is higher for male elderly compared to female counterparts though the difference is not significant.

The high prevalence of morbidity observed in the present study suggests that there is an urgent need to develop geriatric health care services in the developing country like India. Most of the developing countries like India are least prepared to meet the challenges of societies with rapid increase in ageing population [57]. The WHO has recently taken initiatives towards elderly-friendly primary healthcare and has introduced ‘Age-Friendly Primary Health Care Centers Toolkit’ aiming at improving the primary healthcare responses to older persons. Efforts should be made to educate the primary health care workers regarding explicit needs of the elderly and directions should be provided to make the primary health care management more open and friendly to the requirements of the elderly [58].

Since multi-morbidity may cause significant cognitive and functional consequences researcher and policy makers should work together to develop effective intervention strategies and programs to reduce the burden of multi-morbidity. Moreover, new health care model should be developed to meet the health care needs of elderly people with multi-morbidity in India.
